# Microstructure and Mechanical Properties of an Ultrasonic Spot Welded Aluminum Alloy: The Effect of Welding Energy

**DOI:** 10.3390/ma10050449

**Published:** 2017-04-25

**Authors:** He Peng, Daolun Chen, Xianquan Jiang

**Affiliations:** 1 College of Engineering and Technology, Southwest University, Tiansheng Road 2, Beibei District, Chongqing 400715, China; he.peng@ryerson.ca; 2 Department of Mechanical and Industrial Engineering, Ryerson University, 350 Victoria Street, Toronto, ON M5B 2K3, Canada; 3 Faculty of Materials and Energy, Southwest University, Tiansheng Road 2, Beibei District, Chongqing 400715, China; 4 Advanced Materials Research Center, Chongqing Academy of Science and Technology, Yangliu Road 2, Chongqing 401123, China

**Keywords:** aluminum alloy, ultrasonic spot welding, EBSD, microstructure, tensile strength, fatigue

## Abstract

The aim of this study is to evaluate the microstructures, tensile lap shear strength, and fatigue resistance of 6022-T43 aluminum alloy joints welded via a solid-state welding technique–ultrasonic spot welding (USW)–at different energy levels. An ultra-fine necklace-like equiaxed grain structure is observed along the weld line due to the occurrence of dynamic crystallization, with smaller grain sizes at lower levels of welding energy. The tensile lap shear strength, failure energy, and critical stress intensity of the welded joints first increase, reach their maximum values, and then decrease with increasing welding energy. The tensile lap shear failure mode changes from interfacial fracture at lower energy levels, to nugget pull-out at intermediate optimal energy levels, and to transverse through-thickness (TTT) crack growth at higher energy levels. The fatigue life is longer for the joints welded at an energy of 1400 J than 2000 J at higher cyclic loading levels. The fatigue failure mode changes from nugget pull-out to TTT crack growth with decreasing cyclic loading for the joints welded at 1400 J, while TTT crack growth mode remains at all cyclic loading levels for the joints welded at 2000 J. Fatigue crack basically initiates from the nugget edge, and propagates with “river-flow” patterns and characteristic fatigue striations.

## 1. Introduction

The required reduction of climate-changing, environment-damaging, and human death-causing emissions is propelling the transportation industry to improve fuel efficiency [1,2,3,4,5,6]. Vehicle lightweighting is one of the most effective methods to increase fuel efficiency and reduce emissions. Lightweight aluminum alloys have been increasingly used in the automotive industry due to their low density, high specific strength, superior ductility, machinability, recyclability, and environmental friendliness [7,8,9,10,11,12]. The manufacturing of lightweight vehicles inevitably involves welding and joining processes. Welding is indeed an essential aspect of automobile manufacturing, as emphasized by Bhadeshia [13] and Gould [14]. Resistance spot welding (RSW) is a well-established traditional joining technology for steels in auto-body construction. However, when it is used to weld aluminum alloys, some defects (gas pores, cracks, contraction cavity, void, etc.) may occur, leading to unstable weld quality, along with large energy consumption (about 20 kWh per 1000 joints) and short electrode life [10,15,16]. Some other joining techniques have thus been explored, including self-piercing riveting, adhesive bonding, and laser welding. The adhesive bonding and self-pierce riveting would increase the weight of the body structures and the cost of surface treatment [17,18]. Fusion welding techniques like laser welding are limited again due to the presence of potential defects in the weld of aluminum and magnesium alloys [15,19,20,21]. Therefore, solid-state spot welding techniques have been developed to join lightweight aluminum and magnesium alloys. One method is friction stir spot welding (FSSW) [12,16,22,23,24,25,26,27,28,29], and the other is ultrasonic spot welding (USW) [16,30,31]. USW is considered an emerging technology, which is being investigated as a candidate replacement technology for RSW in the welding of aluminum alloys [14], due to its shorter weld cycle (typically <1 s), lower energy consumption, environmental friendliness, and superior joint strength [12,16,22,23,24,25,26].

There are a number of studies on similar and dissimilar solid-state welding of Al alloys, including 2009, 5754, 6061, and 6111 alloys as well as Al/Mg/Al tri-layered clad sheet [15,25,30,31,32,33,34,35,36,37,38,39]. For example, Bakavos and Prangnell [32] studied ultrasonic spot welded (USWed) AA6111-T4 joints along with the joining mechanisms and microstructural changes. The failure load increased with increasing welding energy, a peak load value was reached, and then the failure load decreased with increasing energy. It was observed that the extensive plastic deformation occurred within the weld zone which is not only localized at the interface but develops throughout the entire thickness of the welded sheet [15,25,32,40,41]. A similar trend of changes in the tensile lap shear failure load was reported in [30,35] for the 5754 Al alloy and in [39,42] for the ZEK100 Mg alloy. Chen et al. [38] showed that the deformation at a high strain rate of ~10^3^ s^−1^ during USW of the Al 6111-T4 Al alloy could considerably increase the vacancy concentration at the weld interface, which was estimated to be ~100 times higher than that in a conventionally solution-treated and quenched material. Haddadi and Tsivulas [15] also conducted USW of the AA6111-T4 alloy, and observed that the “folds” or “crests” appeared at a longer welding time and the microstructure and texture varied significantly depending on the location within the weld. Zhang et al. [30], Mirza et al. [33], and Macwan et al. [35] studied the USWed AA5754 alloy joints along with the tensile and fatigue resistance, and they observed the necklace-like structure consisting of fine grain sizes along the weld interface. While the effect of process parameters (vibration amplitude, welding time) on the tensile and fatigue properties of the USWed Al6022 joints was studied [43,44], to the authors’ knowledge, no detailed microstructural evolution, especially via electron backscatter diffraction (EBSD) analysis, is available in the literature. In the present work, a detailed microstructural characterization is performed by using EBSD, with emphasis on the effect of welding energy on the microstructure evolution, grain growth, and grain boundary misorientation. The lap shear tensile properties and fatigue resistance will also be evaluated in relation to the welding energy.

## 2. Materials and Methods

The material used in this study is a 1.3 mm thick sheet of Al6022-T43 alloy, with its nominal chemical composition listed in Table 1. The USW is conducted using a 2.5 kW dual-wedge reed Sonobond MH2016 HPUSW system operated at 20 kHz in an energy mode. The welding parameters used are shown in Table 2. The welding energy (Q) is determined by the level of power (P) and weld time (*t*), where Q = P × t. For example, a welding energy of 1000 J at 2 kW is equivalent to ~0.5 s. The test coupons with dimensions of 80 mm length and 15 mm width are sheared for the lap tensile tests and fatigue tests, where the length direction is parallel to the sheet rolling direction. Prior to welding, the sample surface is ground using 120 grit sand paper, cleaned by ethanol, and dried by compressed air. During USW, the welding tip is positioned at the center of a 20 mm overlap with a vibration direction perpendicular to the rolling direction.

The USWed samples for electron back-scattered diffraction (EBSD) examinations are cut along the weld joints center and parallel to the vibration direction using a slow diamond cutter, and then are cold-mounted using epoxy and mechanically polished with abrasive papers, diamond paste, and colloidal silica. EBSD analyses are carried out at a step size of ~0.8 µm by means of an Oxford integrated AZtecHKL advanced EBSD system with NordlysMax2 and AZtecSynergy along with a large area analytical silicon drift detector. An acceleration voltage of 20 keV, a working distance of 15 mm, and a sample tilt angle of 70° are selected for acquiring the data. AZtecHKL and MTEX software are chosen to analyze the EBSD data. Fairly high indexed EBSD solutions are obtained for all the samples (i.e., 90.0% for the BM; 86.7% for USWed joint made with a welding energy of 1400 J; 79.4% for 800 J). Hence only low noise reduction filters are applied for analyzing the EBSD results. When the EBSD data is rotated, a CS1 data acquisition coordinate system and normal projected maps (IPF-Z) in AZtecHKL software are used. The tensile shear tests are performed by a fully computerized United testing machine at a constant crosshead speed of 1 mm/min in air at room temperature to acquire the maximum failure load. Load-controlled fatigue tests are conducted using a fully computerized Instron 8801 servo-hydraulic testing system at different maximum cyclic loads. A load ratio of *R* (*P*_min_/*P*_max_) = 0.2, a frequency of 50 Hz, and a sinusoidal waveform are selected for the tests. Two spacers with a thickness of 1.3 mm, width of 15 mm, and length of 35 mm are attached at both ends of the specimen to avoid the specimen bending moment and rotation during the tensile and fatigue tests. The fracture surfaces of the selected tensile and fatigue failed samples are examined via SEM to identify fracture mechanisms.

## 3. Results and Discussion

### 3.1. Microstructure Characterization

**Figure 1:**
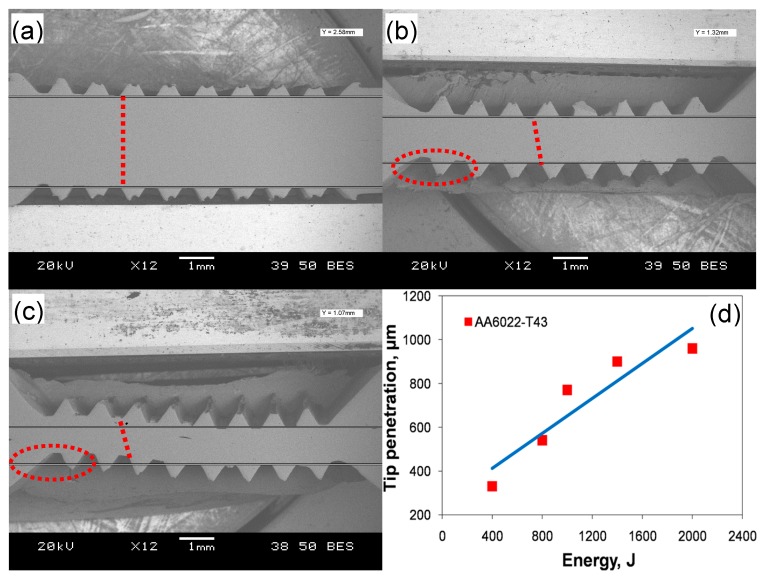
Typical cross-sections of ultrasonic spot welded joints made with a welding energy of (**a**) 800 J; (**b**) 1400 J; (**c**) 2000 J; along with (**d**) Sonotrode welding tip tooth penetration into the aluminum workpiece as a function of the welding energy from 400 J to 2000 J.

Figure 1 shows several typical cross-sections of ultrasonic spot welded joints made with varying levels of welding energy (a–c), and the Sonotrode welding tip tooth penetration as a function of welding energy (d). It is seen that the thickness and pattern of the teeth change with increasing energy. At a lower energy of 800 J (Figure 1a), the tip penetration is shallow and the dent is not fully formed due to the low temperature arising from friction and vibration, which is not enough to generate sufficient plastic deformation because of low energy and short welding time. At this low energy level, the pattern is almost kept in line, i.e., the upper and lower tip tooth dents are oriented vertically as indicated by the vertical dotted line in Figure 1a. Also, the workpieces do not exhibit bending and the reduction in the sheet thickness in-between the welding tips is small at this energy level. With increasing energy to 1400 J and 2000 J (Figure 1b,c), the dents are fully formed due to the materials being softened enough and the penetration being deep. In Figure 1b,c, the red tilted dotted lines and circles show that the sheets become increasingly thinner and the pattern changes to shearing between the welding tip teeth. It means that they are out of phase or out of alignment due to the displacement caused by severe deformation at higher levels of welding energy. A similar phenomenon was also observed in [15,45]. It can be seen from Figure 1c that at higher levels of energy, the pattern changes and bending occurs more obviously, in comparison with Figure 1b. As seen from Figure 1d, the welding tip penetration increases almost linearly with increasing welding energy from a depth of ~330 μm to ~960 μm. Patel et al. [46], Macwan et al. [36,47], and Bakavos and Prangnell [32] pointed out that the welding tip penetration was related to the lap shear strength of the welded joints. With increasing penetration, the microbond density becomes increasingly higher, leading to a higher strength; once the penetration exceeds a threshold, the strength reduces significantly.

**Figure 2:**
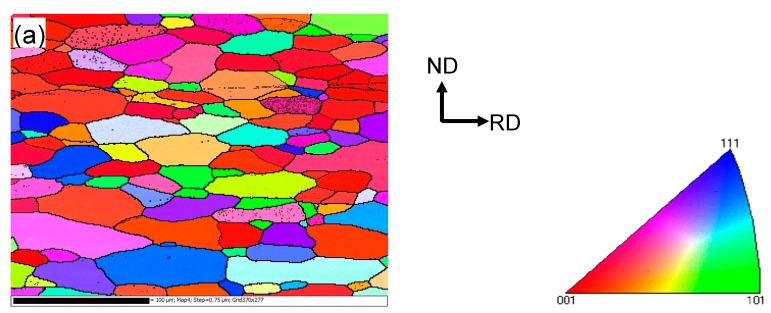

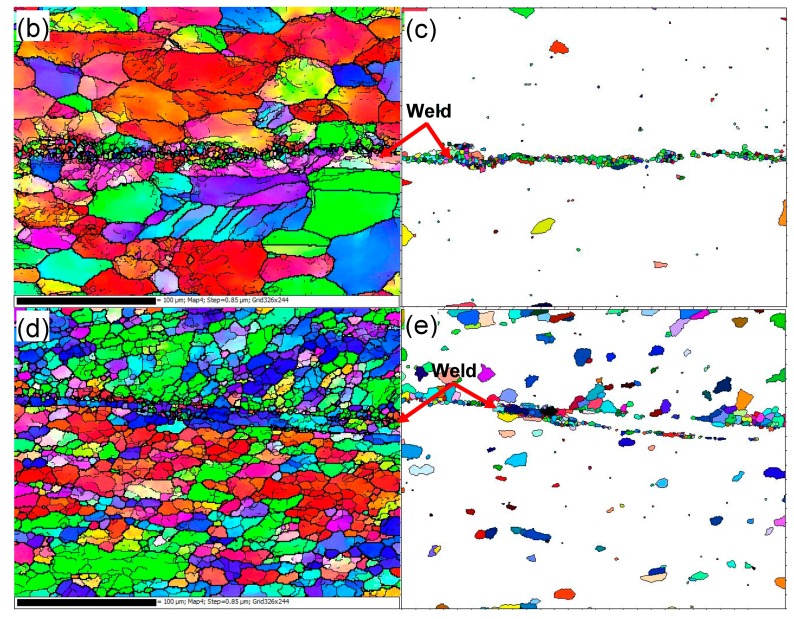
Typical electron backscatter diffraction (EBSD) orientation maps of 6022-T43 Al alloy from (**a**) Base metal (BM); (**b**,**c**) USWed joint made with a welding energy of 800 J; and (**d**,**e**) USWed joint made with a welding energy of 1400 J.

The microstructure in the vicinity of the faying surface in the weld nugget zone (WNZ) changes significantly due to the plastic deformation and heat generated by friction [33,39,42]. Figure 2 presents the EBSD orientation maps of the base metal (BM) and USWed joints made with a welding energy of 800 J and 1400 J, respectively. It should be noted that Figure 2c,e are presented to better see the dynamic recrystallization with increasing welding energy, where the grains with a perimeter less than 50 μm are extracted from Figure 2b,d using the Matlab software (Mtex 3.5.0, Technische Universität Bergakademie, Freiburg, Germany).

It is seen from Figure 2a that the BM has a somewhat deformed grain structure that is elongated along the rolling direction (RD), where the grain size varies between 12–26 μm with an average size of ~19 μm. An equiaxed ultra-fine “necklace”-like grain structure occurs along the welding line, as seen in Figure 2b,c. This is likely due to the occurrence of dynamic recrystallization (DRX) during USW. Similar results were reported in a USWed 5754 Al alloy [33]. The equiaxed grain size along the weld line in Figure 2d,e appears slightly larger due to the higher temperature at a higher level of welding energy. The dynamic recrystallization basically happens along the weld line in the weld nugget zone because of the higher temperatures caused by the more powerful friction and more severe plastic deformation [15,17,32,38,48,49,50]. The equiaxed grain structure in the bulk metal away from the bond interface shown in Figure 2d is smaller than that in Figure 2a,c. This is likely due to the effect of higher temperatures that exceed the recrystallization temperature. Similar results were also reported in an USWed copper alloy [51]. Several other authors also observed the formation of fine grains near the faying surface during USW [15,32,33] and during friction stir welding (FSW) of aluminum alloys [50,52,53,54,55,56]. With an increasing amount of deformation, the dislocation density increases leading to the build-up of internal stored strain energy, which is the driving force for the initiation of recrystallization; the boundary migration and slip contributions are restrained by solute drag so that the lattice rotation develops progressively at grain boundaries. When the deformation reaches a certain degree, the DRX occurs by lattice rotation as new dislocation-free grains form at prior boundaries as initiation sites [32,33,54]. With the development of DRX, the dislocation density and the build-up of stored strain energy will be consumed gradually, and the grains will stop to grow [33,54,57], then the necklace-like structure consisting of ultra-fine grains emerges in the recrystallization initiation sites as shown in Figure 2b–e. Zhang et al. [30] also considered that the formation of the necklace-like structure was related to the mechanical interlocking along the faying surface.

**Figure 3:**
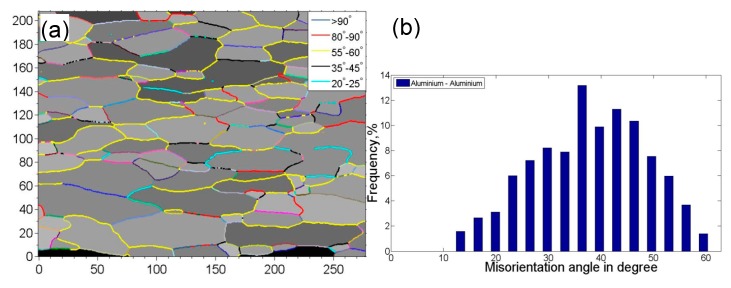

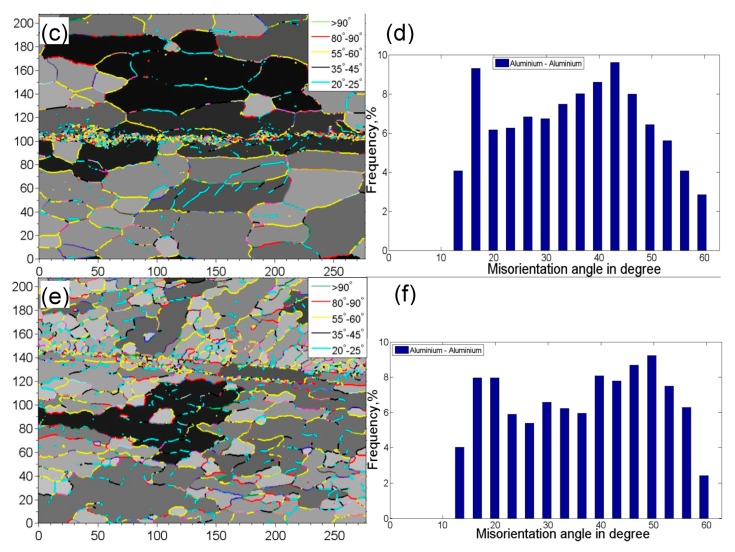
Grain boundary mapping along with the misorientation angle distribution of the 6022-T43 Al alloy. (**a**,**b**) BM; (**c**,**d**) the USWed joint made with a welding energy of 800 J; and (**e**,**f**) the USWed joint made with a welding energy of 1400 J.

Figure 3 shows the grain boundary mapping and misorientation angle distribution of USWed Al6022 Al alloy joints made at the energy of 800 J and 1400 J, respectively, in comparison with those of BM. It is seen that the peak misorientation angle with the highest frequency increases with increasing welding energy, i.e., ~36° in the BM, ~43° for the USWed joint made with an energy of 800 J, and ~50° for the USWed joint made with an energy of 1400 J. The presence of the high-angle grain boundaries is basically related to a large number of ultra-fine equiaxed DRX grains along the weld line with nearly dislocation-free interiors (Figure 2b–e). In other words, when the DRX occurs more fully, more high-angle grain boundaries are expected. Similar results were also reported in [35,38,55].

### 3.2. Lap Shear Tensile Strength and Failure Mode

**Figure 4:**
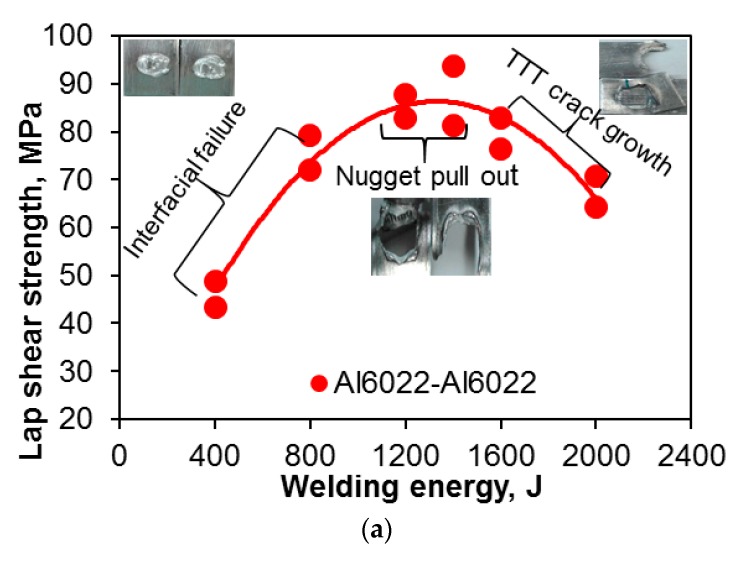

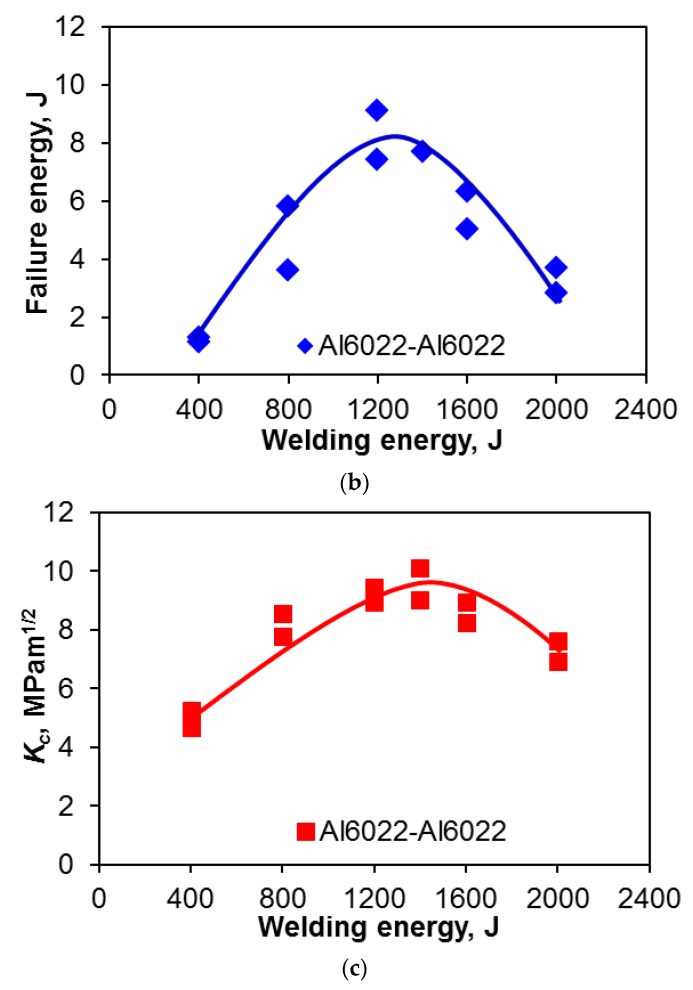
(**a**) The tensile lap shear strength; (**b**) total failure energy; and (**c**) critical stress intensity factor (*K_c_*) of USWed 6022-T43 Al alloy as a function of welding energy at a welding power of 2 kW and a clamping pressure of 0.4 MPa.

Figure 4 presents the maximum tensile lap shear strength, total failure energy, and critical stress intensity factor (*K*_c_) of the USWed 6022-T43 Al alloy as a function of the welding energy at a constant power of 2.0 kW and clamping pressure of 0.4 MPa. The critical stress intensity (*K*_c_) in Figure 4c, which is used to better normalize the effect of the energy input, is calculated based on Zhang’s solution [43,58],

(1) $$K_{c}=0.694\frac{F_{t}}{d\sqrt{t}}$$

where *F_t_* is the peak tensile load, *t* is the sheet thickness and *d* is the nugget diameter. In the present study, the nugget zone area *A* = 8 × 5 mm^2^ is used to calculate shear strength and the equivalent nugget diameter *d* is calculated from,

(2) $$d=\sqrt{\frac{4A}{\pi }}$$

The tensile lap shear strength, total failure energy, and critical stress intensity have a similar trend, i.e., they first increase with increasing welding energy up to 1400 J, and then decrease with further increasing energy. It is known that with increasing temperature, the yield strength of materials decreases, which determines the density or extent of microbonding through metallurgical adhesion and mechanical interlocking during USW [32,34,39,47,59]. The temperature and strain rate are relatively low at lower energy inputs such as 400 J and 800 J (Figure 4). In this stage, the strength of the USWed joints is mainly determined by the net microbond area [15,32,34,39,47,59]. The material is not softened enough, and the flowability of metal is limited, leading to a relatively low microbond density, a relatively low strength of 46–76 MPa, a relatively low total failure energy of 1.2–4.7 J, and a relative low critical stress intensity of 4.9–8.0 MPam^1/2^ for 400 J and 800 J, respectively.

Interfacial failure is also observed at the welding energies of 400 J and 800 J, as indicated in Figure 4a. A more complete coalescence of the metallic interface occurs with increasing temperature at a higher level of welding energy due to the higher strain rate and more severe plastic deformation [43,60], and the failure mode changes from the interfacial failure to nugget pull-out, when the welding energy increases to 1200 J. As reported in [15], the highest temperature during USW appeared at the edge of the welding tip. Then the first softened place will be at the nugget edge, and the highest pressure caused by the elastic deflection of the sheets occurs there as well as the initial bonding, so the stress concentration at the nugget edge occurs [15,61]. The highest tensile lap shear strength of ~94 MPa (corresponding to the maximum load of ~3.8 kN, the total failure energy of ~14.1 J, and the critical stress intensity of ~10.1 MPam^1/2^) happens at the energy of 1400 J.

It suggests that the microbond density at the energy of 1400 J reaches a high level because more extensive plastic deformation and higher temperature makes the materials more softened, with the microbonding developed and coalesced more thoroughly. In this case, the effect of stress concentration at the nugget edge is less than that of the microbond density on the tensile lap shear strength. It should be noted that the welding energy required to reach a maximum strength is different for different materials, material states, and sample thickness [15,25,30,32,33,42]. It is worthwhile to note that the maximum tensile lap shear load (~3.8 kN) is higher than that (3.43 kN) of the 1.2 mm thick Al6022-T4 alloy joints via friction stir spot welding [62]. Beyond the welding energy of 1400 J, the tensile lap shear strength, total failure energy, and critical stress intensity (*K_c_*) all decrease with increasing energy due to the presence of a higher stress concentration caused by the deeper tip penetration (Figure 1). As indicated in Figure 4a, the failure mode changes to transverse through-thickness (TTT) crack growth because of the higher stress concentration present at the nugget edge caused by the higher temperature and deformation [15,34,39,47,59]. The reduced material thickness as indicated in Figure 1, along with further better microbonding of the faying surface, is another key factor of producing such a TTT failure mode [34,35,39,46,47,53,59,62].

### 3.3. Fractography: Tensile Lap Shear Fracture Surface Examination

**Figure 5:**
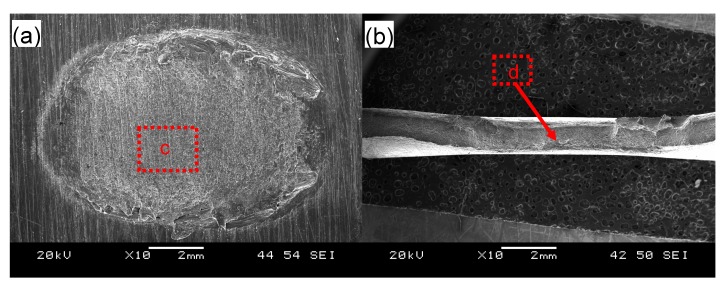

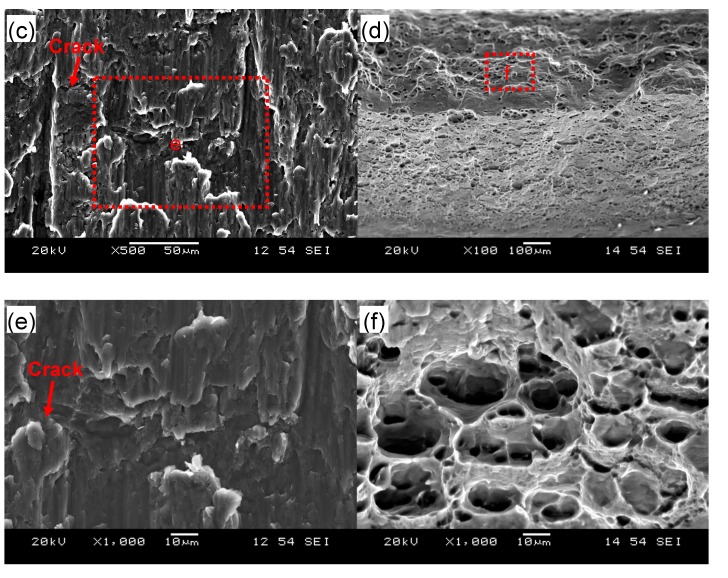
Typical SEM images of the tensile lap shear fracture surfaces of USWed joints of the 6022-T43 Al alloy made with a welding energy of 800 J (**a**,**c**,**e**) and 2000 J (**b**,**d**,**f**); with (**a**,**b**) showing an overall view; (**c**,**d**) at a lower magnification; and (**e**,**f**) at a higher magnification.

Figure 5 shows typical fracture surface images of the USWed 6022-T43 Al alloy joints made with a welding energy of 800 J and 2000 J, respectively. It is seen that interfacial failure in between two welded sheets occurs at a lower energy of 800 J (Figure 5a), while TTT crack growth appears at a higher energy of 2000 J (Figure 5b). In the mode of interfacial failure, larger deformation appears at the nugget edge in comparison with the nugget center (Figure 5a); this corroborates well the plastic deformation-induced high temperature during USW that occurs at the nugget edge than at the nugget center [15,32,34,38,39,63,64]. From the magnified images of the joint welded at an energy of 800 J (Figure 5c,e), typical shear deformation characteristics with shear ridges are observed, along with some secondary cracks that are considered to be the initiation sites of fracture, as indicated by the arrows. It suggests that the BM reaches its shear fracture strength at some local places [42]. Scrubbing lines along the shear loading direction are also observed in Figure 5c,e. This is attributed to the abrasion or galling of asperities of the microscale roughness of the fracture surfaces and the ground sheet surfaces during the tensile lap shear testing of the USWed joints [33]. In the failure mode of TTT crack growth at higher energy levels, the characteristic dimple fracture features are observed in the magnified images in Figure 5d,f, indicating the distinctive ductile fracture consisting of microvoid formation/nucleation, growth, and coalescence in the 6022-T43 Al alloy [30,33].

### 3.4. Fatigue Behavior and Failure Mode

**Figure 6:**
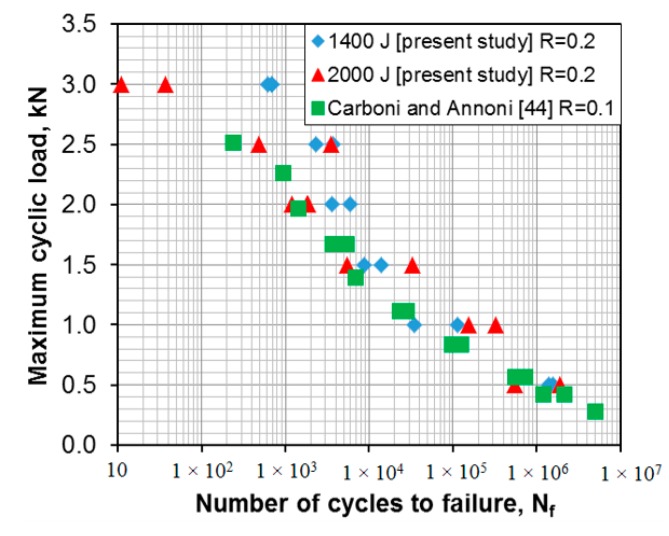
Fatigue life curves of the USWed 6022-T43 Al alloy joints made at a welding energy of 1400 J and 2000 J, respectively, and tested at RT, *R* = 0.2, and a frequency of 50 Hz.

Fatigue tests of the USWed 6022-T43 Al alloy joints made with a welding energy of 1400 J and 2000 J, respectively, were performed at a load ratio of *R* = 0.2, a frequency of 50 Hz, and at room temperature (RT), and the obtained results are plotted in Figure 6. For the sake of comparison, the fatigue data of the USWed 6022-T4 Al alloy joints obtained at *R* = 0.1 [44] are plotted in the figure as well. It is seen that at lower cyclic load levels, the fatigue life of the presented USWed joints is in agreement with that reported in the literature. At higher cyclic load levels, the fatigue life of the presented USWed joints made with a welding energy of 1400 J is indeed longer. With respect to the effect of the welding energy, at the higher maximum loads of 2–3 kN, the 1400 J samples show a longer fatigue life compared with the 2000 J samples. This corresponds well to the tensile lap shear test results shown in Figure 4, where the 1400 J samples have a higher tensile lap shear strength than the 2000 J samples. This is understandable since in the low-cycle fatigue regime below ~10^4^ cycles, the situation is closer to the quasi-static tensile tests due to the occurrence of plastic deformation at higher applied loads. 

**Figure 7:**
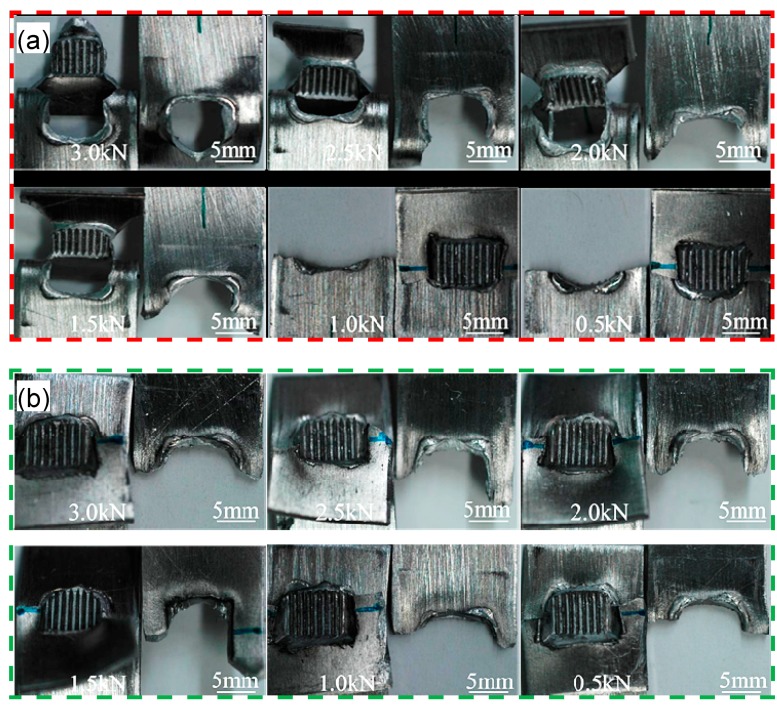
Macroscopic images showing the fatigue failed samples of the USWed 6022-T43 Al alloy joints tested in the conditions of (**a**) 1400 J-(3.0–0.5) kN and (**b**) 2000 J-(3.0–0.5) kN.

Figure 7a,b shows the macroscopic images of the fatigue failed samples at the maximum cyclic load from 3.0 to 0.5 kN, which are welded at an energy of 1400 J and 2000 J, respectively. 

As seen in Figure 7a, the nugget pull-out failure mode is observed at the higher cyclic load levels (1.5–3.0 kN), while the transverse through-thickness (TTT) crack growth mode is observed at the lower cyclic load levels (0.5–1.0 kN). This is in agreement with the report by Roesler et al. [64] and Xu et al. [65,66] who pointed out that the dynamic fatigue property is much more sensitive to the various factors of materials and the manufacturing process (e.g., local stress concentration, severe weld concavity, residual stresses, surface conditions, surface protective coating, and zinc inclusions in weldment) at the lower cyclic load level than the higher cyclic load level. During USW at a higher energy input, a small micro-level notch tip that experiences a higher stress concentration caused by the higher temperature and more severe deformation at the notch of two sheets occurs, and the stress concentration leads to the initiation and propagation of fatigue cracks perpendicular to the loading direction [32,33,42,46,61]. Once the dynamic stress which acts on the remaining cross section exceeds a threshold, the transverse through-thickness crack growth occurs at the severely deformed nugget edge which is related to the welding tip [15,32,33,46]. From Figure 7b it can be seen that the transverse through-thickness crack growth basically occurs at all cyclic load levels applied from 3.0 kN to 0.5 kN. This is attributed to the fact that the 2000 J USWed samples underwent a higher stress concentration than the 1400 J USWed samples, which is caused by the bigger thickness change at the nugget edge in conjunction with the sample bending deformation [32,46,58].

**Figure 8:**
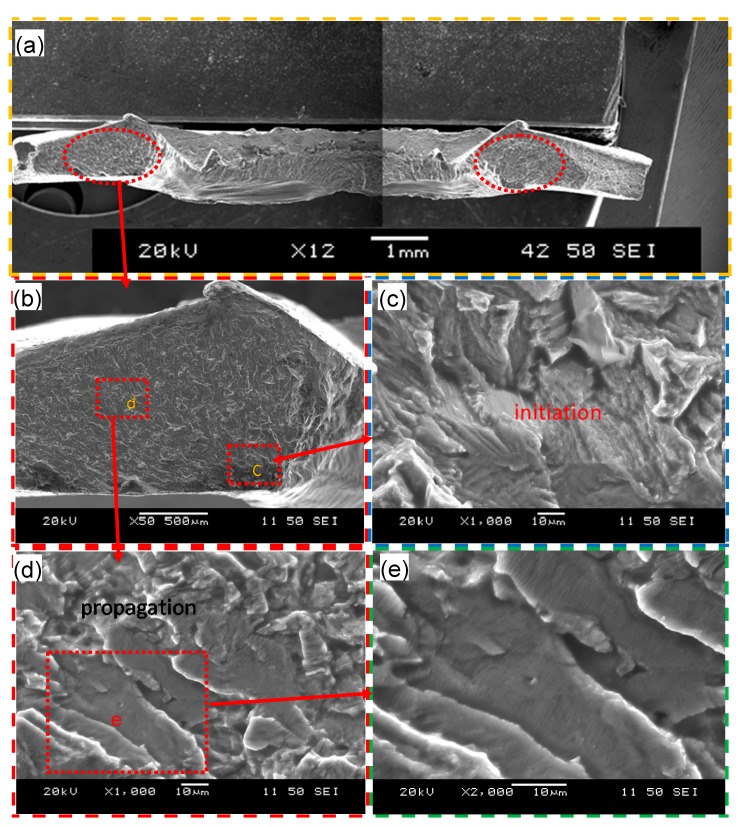
Typical SEM images of a fatigue failed sample made at a welding energy of 1400 J and tested at a maximum load of 0.5 kN. (**a**) Overall view of the entire fracture surface and (**b**–**e**) magnified view of the regions of interest indicated in (**a**,**b**).

Typical SEM images of the fatigue fracture surface of a failed USWed joint welded at an energy of 1400 J and tested at a maximum cyclic load of 0.5 kN are shown in Figure 8, with an overall view of the entire fracture surface in Figure 8a. A similar or nearly symmetric character in two red-circled zones on the left and right sides of the image can be seen. One of the red-circled zones is magnified, and the regions c and d are further magnified in the Figure 8c,d. It is clear that the fatigue crack initiated from the corner near region c (Figure 8b,c), and then the fatigue crack propagated along fan-shaped divergent directions with fairly well-defined “river-flow” patterns. This corroborates well the presence of stress concentration at the corner near region c, i.e., the nugget edge as mentioned above. Similar observations were also reported by Patel et al. [46] in ultrasonic spot welded Mg alloy joints. From the further magnified images in Figure 8d,e, it is seen that the fatigue crack propagation is basically characterized by the characteristic fatigue striations with a direction perpendicular to the fatigue crack propagation direction. During fatigue tests especially at the low-cycle and high-load levels, the repeated plastic blunting-resharpening process in the face-centered cubic materials, like the current Al6022-T43 alloy, will result in the formation of fatigue striations which stem from the glide of dislocations on the slip plane along the slip direction in the plastic zone ahead of the fatigue crack tip [26,33].

## 4. Conclusions

Ultrasonic spot welding of the Al6022-T43 alloy was conducted at different levels of welding energy, and the microstructure, tensile lap shear strength, and fatigue life were evaluated. The following conclusions can be drawn.

(1) An ultra-fine “necklace”-like equiaxed grain structure is observed along the weld line as a result of the occurrence of dynamic crystallization. The equiaxed grain size along the weld line appears slightly larger at a higher level of welding energy due to the higher temperature.
(2) High-angle grain boundaries are present in both the base metal and ultrasonic spot welded (USWed) joints, and the misorientation angles of the grain boundaries increase with increasing welding energy.
(3) A tensile lap shear strength as high as ~94 MPa is achieved at a welding energy of 1400 J. As the welding energy increases, the tensile lap shear strength, failure energy, and critical stress intensity factor of USWed joints first increase, reach their maximum values, and then decrease.
(4) The failure mode in the tensile lap shear tests is observed to be interfacial fracture at the lower energy levels, nugget pull-out at the intermediate optimal energy levels (i.e., 1200–1400 J), and transverse through-thickness (TTT) crack growth at the higher energy levels due to the presence of stress concentration at the nugget edge.
(5) While fatigue life of the USWed joints made at the energy levels of 1400 J and 2000 J is equivalent within the experimental scatter at the lower cyclic loads, the joints made at 1400 J exhibit a longer fatigue life at the higher cyclic loading levels.
(6) The fatigue fracture mode changes from nugget pull-out to TTT crack growth with decreasing cyclic loading levels for the USWed joints made at 1400 J, while TTT crack growth mode occurs at all cyclic loading levels for the USWed joints made at 2000 J.
(7) Fatigue crack is observed to initiate from the nugget edge due to the presence of stress concentration, and propagate along fan-shaped divergent directions with some “river-flow” patterns, exhibiting characteristic fatigue striations perpendicular to the fatigue crack growth direction.

## Figures and Tables

**Table 1:** Chemical composition of the Al6022-T43 alloy.

Elements	Zn	Si	Cr	Fe	Mg	Cu	Mn	Ti	Al
wt %	0.2	1.0	0.25	0.5	1.0	0.1	0.7	0.1	Bal.

**Table 2:** Welding parameters selected in the present study.

Ultrasonic Power	Clamping Pressure	Welding Energy	Welding Time	Impedance Setting	Frequency
2000 W	0.40 MPa	400–2000 J	0.2–1 s	1	20 kHz
